# Co-expression of RNA–protein complexes in *Escherichia coli* and applications to RNA biology

**DOI:** 10.1093/nar/gkt576

**Published:** 2013-06-25

**Authors:** Luc Ponchon, Marjorie Catala, Bili Seijo, Marguerite El Khouri, Frédéric Dardel, Sylvie Nonin-Lecomte, Carine Tisné

**Affiliations:** ^1^CNRS, UMR 8015, Laboratoire de Cristallographie et RMN biologiques, 4 avenue de l’Observatoire, 75006 Paris, France and ^2^Université Paris Descartes, Sorbonne Paris Cité, UMR 8015, Laboratoire de Cristallographie et RMN biologiques, 4 avenue de l’Observatoire, 75006 Paris, France

## Abstract

RNA has emerged as a major player in many cellular processes. Understanding these processes at the molecular level requires homogeneous RNA samples for structural, biochemical and pharmacological studies. We previously devised a generic approach that allows efficient *in vivo* expression of recombinant RNA in *Escherichia coli*. In this work, we have extended this method to RNA/protein co-expression. We have engineered several plasmids that allow overexpression of RNA–protein complexes in *E. coli*. We have investigated the potential of these tools in many applications, including the production of nuclease-sensitive RNAs encapsulated in viral protein pseudo-particles, the co-production of non-coding RNAs with chaperone proteins, the incorporation of a post-transcriptional RNA modification by co-production with the appropriate modifying enzyme and finally the production and purification of an RNA–His-tagged protein complex by nickel affinity chromatography. We show that this last application easily provides pure material for crystallographic studies. The new tools we report will pave the way to large-scale structural and molecular investigations of RNA function and interactions with proteins.

## INTRODUCTION

In the past decade, novel roles and applications have been uncovered for RNAs in a variety of fields [reviewed in (1)], namely, microRNAs, aptamers, ribozymes, riboswitches (2), mRNA splicing and translation, control of virus replication and nano-object fabrication (3,4). Many newly discovered functions of RNAs are regulatory mechanisms. RNAs can regulate many vital cellular processes in eukaryotes, some of which are associated with cancers (5–7) and neurodegenerative pathologies (8,9). As a consequence, RNAs are thus promising therapeutic targets [reviewed in (10)]. Understanding such processes at molecular and atomic levels requires structural, biochemical and pharmacological investigations and, therefore, large amounts of homogeneous RNA. Systematic studies of RNA structure and function or screening of RNA-binding ligands in drug development usually requires milligram quantities of homogeneous and pure RNA. As opposed to proteins, the study of RNA is still hampered by difficulties in obtaining large quantities of pure samples. To date, RNA has mostly been produced *in vitro* either by transcription using T7 RNA polymerase or by chemical synthesis. Recombinant approaches for the production of stable and structured RNA *in vivo* have progressively emerged to produce stable structured RNA *in vivo* [reviewed in (11)]. To date, many RNAs have been produced in *Escherichia coli* using strategies that are based on the production of either a naturally stable RNA or a derivative of such a stable RNA, for instance tRNAs (12–15), RNAse P RNA (16), transfer-messenger RNA (tmRNA) domains (17) or rRNA variants (18). The success of these productions relies on the fact that these RNAs are recognized by cellular machinery, processed precisely, post-transcriptionally modified and not subjected to 3′ polyadenylation, which triggers RNA degradation (11,12,19).

Our group previously devised a generic approach to produce a much larger variety of recombinant RNAs in *E. coli* using a strategy relying on tRNA–RNA fusions (12,19). A ‘tRNA scaffold’ recognized by *E. coli* cellular factors was built from a tRNA acceptor stem, TΨC- and D-arms. The anticodon stem-loop was modified into an insertion region with restriction sites that can accommodate the desired RNA sequence. The insertion region was designed to maintain a hairpin structure. Our method has provided a set of tools for the expression of recombinant RNAs equivalent to those currently available for proteins. More than 50 RNA constructs have been successfully expressed (12–20). A similar strategy was also developed using 5 S rRNA as a fusion scaffold (21). These RNA-scaffold strategies were the first steps toward challenging co-expression studies, where components of a ribonucleic complex could be produced *in vivo*.

Here, we present a new strategy to perform RNA–protein co-expression using *E. coli* as well as a set of tools for rapid purification of intact RNAs and RNA–protein complexes. The potential of such methods has been investigated, and several applications of RNA–protein co-expression are presented. We validate our new co-expression system with the introduction of a post-transcriptional modification—that cannot naturally be performed by *E. coli*—by a recombinant protein on the co-expressed recombinant tRNA. We then present a new methodology that can be used for rapid purification and/or for stabilizing recombinant RNA constructs. This approach is derived from the armored RNA technology (22), which allows the packaging of the RNA constructs into virus-like particles (VLP). As a proof-of-concept, we present the production of a large RNA of 347 nucleotides (nt) (tmRNA) that was sensitive to nucleases when overproduced alone in *E. coli*. Finally, we show that we can produce an Hfq-bound transcript not embedded in the tRNA scaffold, taking advantage of the protection brought by the RNA chaperone against nucleolysis. Lastly, we investigate the potential of co-expressing protein–RNA complexes for structural studies.

## MATERIALS AND METHODS

### Plasmid design

DNA oligonucleotides were purchased from Eurogentec. The second T7 promoter of the pACYCDuet-1 (Novagen) was substituted by a tac promoter between the BsrG1 and Nde1 restriction sites. The resulting plasmid was named pACYCT2 and the protein of interest was subcloned downstream of the tac promoter. All plasmids for RNA production were derived from the pBSTNAV one by assembling synthetic overlapping oligonucleotides as already described (23–26). To construct the pProRNA plasmid, we amplified the *lacI* gene and the cloning region of the pACYCDuet-1 by PCR and inserted the PCR product in the Xho1 site of the pBSTNAV plasmid. The p44K plasmid, which is derived from the pUC18 vector (27), harbors a *lac* promoter, multiple cloning sites encompassing the gene coding for the tRNA scaffold, an rrnc terminator, a T7 terminator, the pMB1 origin and the AmpR coding sequence. The sequences of the cloning/expression region of the different plasmids are available in Supplementary Figure S1.

The armored tRNA scaffold plasmid (pBSTNAV-AtRNA) uses the human tRNA^Lys^_3_ for the tRNA scaffold as previously described (12). The MS2 operator hairpin was cloned between the EagI and SacII restriction sites of the pBSKrna plasmid (19), and the resulting RNA was named AtRNA (Figure 2). This vector was subsequently used to insert the various DNA oligonucleotides coding for the RNA fragments between the AatII and XbaI restriction sites (Figure 2A). Sequences encoding for either the MS2 coat protein (GenBank: AAA32260.1) or the SmpB protein (GenBank: AAA79790.1) were subcloned in the pACYCT2 between the NdeI and XhoI restriction sites.

The gene encoding *E. coli* tmRNA (GenBank: AP009048.1) or the *Aquifex aeolicus* tmRNA coding gene (GenBank: AE000657.1) with the MS2 operator (AtmRNA) was subcloned in the pBSTNAV plasmid between the EcoRI and PstI restriction sites. To overexpress the MS2 coat protein fused to a six-histidine tag, we adopted a previously reported strategy (28) with an internal tag, which was designed to maintain the oligomerization state of the protein. The synthetic DNA for AtmRNA was purchased from GeneCust.

The genes coding for the MS2 coat protein or the AtmRNA were subcloned in the pProRNA plasmid between the NdeI and AatII restriction sites or the EcoRI and PstI restriction sites, respectively. The gene encoding *Thermus thermophilus* TrmI (29) (GenBank: AJ516007.1) was inserted in the p44K plasmid between the NdeI and KpNI restriction sites. The gene encoding human tRNA^Lys^_3_ (26) (GenBank: U00939.1) was inserted between the EcoRI and PstI restriction sites. The gene encoding *E. coli* Hfq (GenBank: ACE63256.1) was inserted in the p44K plasmid between the NdeI and KpNI restriction sites and the *E. coli* SgrS coding gene (GenBank: CP002291.1) between the EcoRI and PstI restriction sites. The cloned fragments of all constructs were checked by DNA sequencing (Millegen, France).

The plasmids have been deposited in Addgene.

### Protein–RNA expression

JM101 or XL1-Blue (Stratagene) *E. coli* strains were used in all expression tests. The use of these strains was crucial to maintain a high expression level from the constitutive *lpp* promoter. The protein expression was induced in the exponential phase (absorbance at 660 nm between 0.6 and 1) or in the first few hours of the stationary phase, by addition of 1 mM isopropyl-l-thio-d-galactopyranoside (IPTG) for 3 h. The analysis of the expression levels was performed from RNA ‘minipreps’ on 5-ml cultures grown in Luria Broth (LB) medium. Cells were pelleted and resuspended in 180 µl of a Tris-HCl buffer (pH 7.4) containing 10 mM magnesium acetate. The suspensions were extracted with water-saturated phenol (200 µl) followed by ethanol precipitation of the aqueous phase. The contents were analysed by SDS–PAGE and RNA bands visualized by UV shadowing or SYBR Safe staining (Invitrogen).

### tRNA^Lys^_3_ methylation

Modified species of tRNA^Lys^_3_ were purified as previously described (26). Briefly, total RNAs were recovered from cells by phenol extraction and loaded on a Resource Q column (GE Healthcare, 50 ml) previously equilibrated in 20 mM potassium phosphate (pH 6.5). The m^1^A_58_ tRNA^Lys^_3_ eluted at 400 mM NaCl, whereas the non-methylated tRNA^Lys^_3_ eluted between 410 and 460 mM.

The protocol used for *in vitro* methylation of tRNA^Lys^_3_ was adapted from a previously reported protocol (29). Briefly, the reaction mixture (2 ml) was composed of 50 mM Tris-HCl (pH 8.0), 10 mM MgCl_2_, 5 mg of tRNA^Lys^_3_, 1 mM AdoMet (*S*-adenosyl-l-methionine) and 450 µg of purified TrmI (30). After 60 min of incubation at 60°C, the reaction was stopped by phenol extraction and ethanol precipitation. The methylated tRNA^Lys^_3_ was purified on a Resource Q column (GE Healthcare, 50 ml) equilibrated in 20 mM potassium phosphate (pH 6.5) and eluted with a gradient of NaCl. The eluate was then extensively dialyzed against 10 mM potassium phosphate (pH 6.5) and 50 mM KCl. NMR experiments were recorded at 288 K on a Bruker AVANCE 600-MHz spectrometer equipped with a TCI 5-mm cryoprobe. Two dimensional NOESY experiments with a mixing time of 150 min were recorded using a watergate sequence for the solvent signal suppression (31).

### RNA–Protein purification

#### Size exclusion chromatography

After induction, bacteria co-expressing the AtRNA/MS2 coat protein were pelleted by centrifugation and lysed by ultrasonic disruption in buffer A (50 mM Tris-HCl buffer, pH 7.5, 300 mM sodium chloride). After centrifugation (20 000× *g* for 10 min), the supernatant was incubated with Benzonase nuclease (Sigma) (100 U/g of bacteria) at 37°C for 1 h. The supernatant was then loaded onto a Superose 6 prep grade (GE Healthcare) equilibrated with buffer A. Fractions were analysed by SDS–PAGE stained with SYBR Safe and Coomassie Brilliant Blue.

#### Affinity purification

Bacteria co-expressing the AtRNA/His6-MS2 coat protein were lysed by sonication in buffer B (50 mM Tris-HCl buffer, pH 7.5, 150 mM sodium chloride) at 4°C. The supernatant was loaded onto a column of Ni-NTA resin equilibrated with buffer B. The column was washed with buffer B supplemented with 20 mM imidazole-HCl until a stable baseline is reached, and then the RNA–protein complex was eluted with a linear gradient over 10-column volumes with buffer B supplemented with 500 mM imidazole-HCl. The fractions were analysed by SDS–PAGE stained with SYBR Safe and Coomassie Brilliant Blue.

#### Anion exchange chromatography

For electron microscopy, the AtRNA/MS2 coat protein complex purified by size exclusion chromatography as previously described was loaded onto an anion exchange column (50 ml, Source 15Q, GE Healthcare). The complex was eluted with a gradient of NaCl between 300 mM and 700 mM in a Tris-HCl buffer (50 mM, pH 8.0) for 10-column volumes.

### Benzonase treatment

One gram of bacteria co-expressing the AtRNA–RNA fusion and the MS2 coat protein (tagged or not) was lysed by sonication in 10 ml of buffer B at 4°C. The supernatant was incubated with 100 U of Benzonase nuclease (Sigma) at 37°C. The RNA was extracted with water-saturated phenol; the aqueous phase was NaCl/ethanol precipitated (0.1 volume of 5 M NaCl and 2.5 volumes of ethanol). The RNA contents were analysed by SDS–PAGE. Bands were revealed by UV shadowing.

### Electron microscopy

After anion exchange chromatography, the AtRNA/MS2 coat protein assembly was adsorbed on carbon/Formvar-coated grids and negatively stained with 1% uranyl acetate. The samples were examined in a JEOL JEM-100 S at 100 kV and at a screened magnification of 100 000×. Imaging was performed at the Cellular and Molecular Imaging facility of the IMTCE (Faculté de Pharmacie, Université Paris Descartes).

### Crystallization and diffraction data collection

After Ni-NTA agarose and size exclusion chromatography, the AtRNA/His6-MS2 coat protein complex was concentrated to 4 mg/ml. Crystallization assays were performed at 4°C using the vapor diffusion method in sitting-drop. The protein–RNA samples were prepared in a 25 mM Tris-HCl buffer (pH 8.0) containing 150 mM or 300 mM NaCl. Drops of 0.5 μl were prepared using a Cybi-Disk robot system that mixes equal volumes of protein and reservoir solutions. Reservoir volumes of 100 μl were used. Crystals were obtained in 1.26 M sodium phosphate monobasic monohydrate, 0.14 M potassium phosphate dibasic (pH 5.6) (Hampton Research - Index kit). The crystals were harvested and flash-frozen in liquid nitrogen before data collection. Diffraction data were collected on beamline ID23-2 of the European Synchrotron Radiation Facility (Grenoble, France).

## RESULTS

### Plasmid design for RNA–protein co-expression using the ‘tRNA scaffold’

In our previous ‘tRNA scaffold’ strategy, the tRNA–RNA fusion (19) was cloned into a high-copy vector derived from the pBluescript KS+ one named pBSTNAV (23). This vector was originally used to produce tRNAs in *E. coli* (23,25,26) and harbors a ColE1 origin of replication. High-copy plasmids bring significant advantages in terms of RNA production yields (11). We thus decided to keep high-copy vectors for the design of new plasmids dedicated to RNA–protein co-expression. Moreover, in pBSTNAV, the lpp promoter upstream of the gene, one of the strongest natural *E. coli* promoters that controls the transcription of the mRNA of the lipoprotein (32), allows production of high levels of recombinant transcripts. Accumulation of stable recombinant RNA throughout the transcription phase can be achieved by the combined use of a strong promoter and a multicopy plasmid. Yields reach a maximum within the first few hours of the stationary phase. Although all *E. coli* strains can be used to express RNA, optimum yields were obtained in *recA* and *endA E.coli* strains, such as DH5 *α*, XL1 or JM101. However, as these strains also do not produce the T7 RNA polymerase, they are not compatible with pET vectors (Novagen), which are often used to express recombinant proteins in *E. coli*.

With the purpose of co-expressing a protein together with a tRNA–RNA fusion in *E. coli*, we first designed a new plasmid compatible with pBSTNAV and dedicated to protein production. In a two-plasmid system, the origins of replications determine the compatibility of plasmids, i.e. their abilities to replicate in conjunction with each other within the same bacterial cell. Plasmids that use the same replication system cannot co-exist in the same bacterial cell. We thus chose to subclone the different protein-coding genes in a pACYC-type vector, its P15A origin of replication being compatible with the pBSTNAV ColE1 origin (33,34). We started with the pACYCDuet-1 vector (Novagen) designed for the co-expression of two target protein genes. This vector contains two multiple cloning sites, each of which is preceded by a T7 promoter/*lac* operator and a ribosome binding site. The recombinant protein was cloned downstream of the second T7 promoter. The pBSTNAV and the pACYDuet-1 vectors contain, respectively, the ampicillin and chloramphenicol resistance genes, allowing selection of bacteria only co-transformed by the two plasmids. The induction of the protein expression is under the control of the *lac* operator and is thus triggered by the addition of IPTG. Although pBSTNAV and pACYCDuet-1 are compatible, our first attempts to obtain co-expression of, respectively, the RNA and the protein in *E. coli* [BL21 (DE3) strain] were unsuccessful. It was impossible to obtain simultaneous expression of both RNA and protein. The protein alone was recovered after 3 h of induction followed by cell lysis, whereas after one night of induction, only the overexpressed RNA was detected.

**Figure 1.**
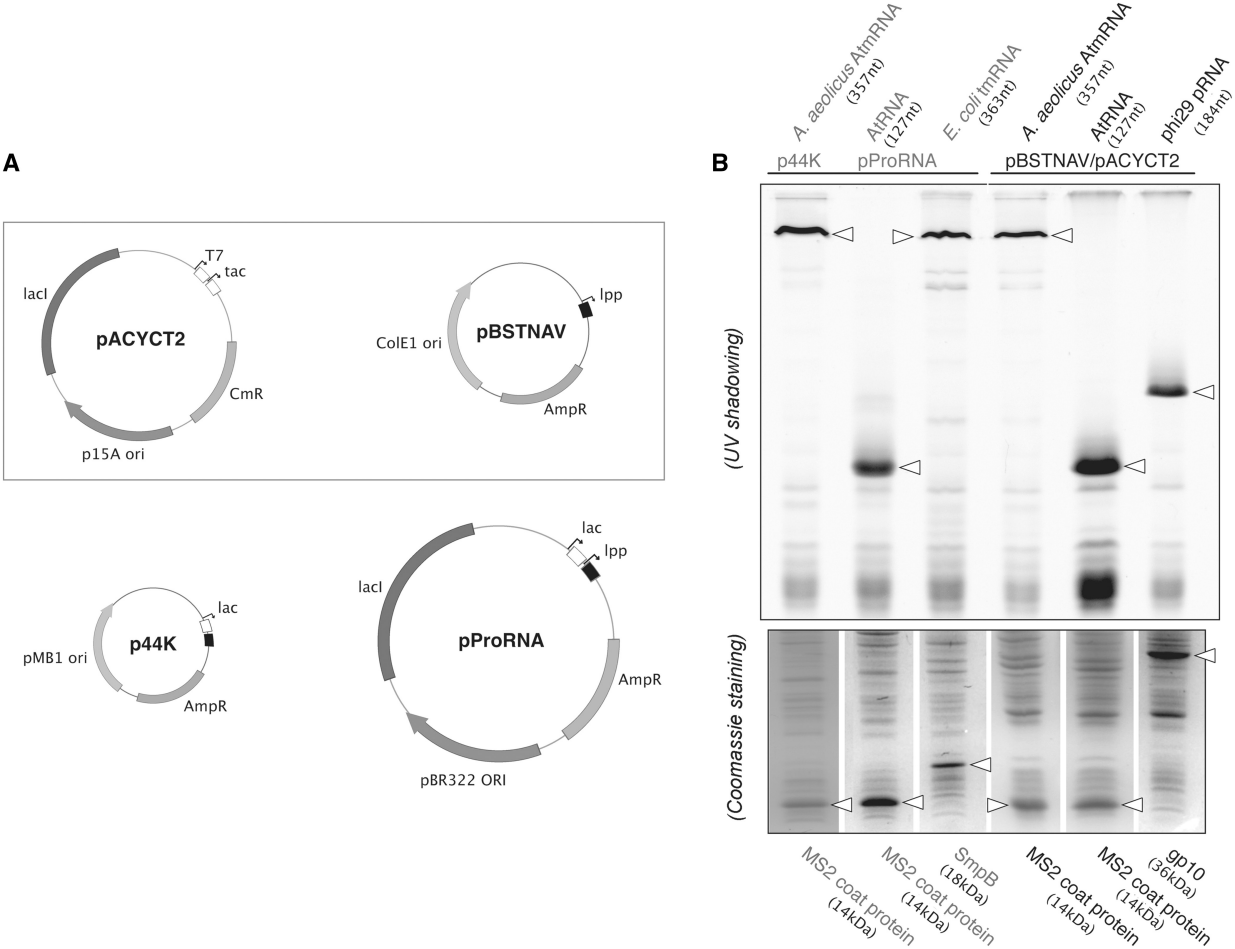
(**A**) Design of the pACYCT2, pBSTNAV, p44K and pProRNA plasmids described in this study. The pBSTNAV and pACYCT2 plasmids are used to co-express RNA–protein pairs using the two-plasmid strategy. (**B**) Co-expression of RNA–protein partners was tested for the *A. aeolicus* AtmRNA/MS2 coat protein, AtRNA/MS2 coat protein, *E. coli* tmRNA/SmpB and phi29 pRNA/gp10 in 5 ml of culture. The ‘A’ before AtmRNA and AtRNA refers to armored RNA. RNA–protein partners were expressed into the two plasmids pBSTNAV and pACYT2 system, the pProRNA system or the p44K plasmid system. The expression levels of RNA (upper gel) following phenol extraction and of total protein in crude extracts (lower gel) were analysed by SDS–PAGE. The RNA bands are visualized by SYBR Safe staining and the protein bands by Coomassie Blue staining. The white triangles indicate the overexpressed RNA and protein for each pair.

To synchronize the RNA and the protein production, we substituted the second T7 promoter in the pACYCDuet-1 vector by the bacterial tac promoter (35). The resulting vector was called pACYCT2 (Figure 1). The protein-coding gene was then inserted downstream of the tac promoter. This new plasmid allows successful co-expression of both the RNA and the protein. We subsequently validated the two-plasmid co-expression strategy with three examples of RNA–protein co-expression assays in *E. coli* (Figure 1). A small RNA of *Bacillus subtilis* bacteriophage phi29 was shown to have an essential role in viral DNA packaging *in vitro* (36). The phi29 pRNA was cloned into the pBSTNAV vector, whereas the gp10 protein that selectively binds to it (37) was cloned into the pACYCT2 vector. As shown in Figure 1, good levels of expression of both pRNA and gp10 were obtained 3 h after addition of 1 mM IPTG in exponential phase of cell growth. The remaining two examples deal with the co-expression of the MS2 coat protein with, respectively, the AtRNA-mala, a tRNA–RNA fusion of 127 nt, and the AtmRNA (Supplementary Figure S2). High levels of expressions of both RNA and protein were observed. Therefore, these three examples validate the feasibility of co-expressing RNA and protein in *E. coli* using two plasmids.

Thereafter, we aimed to design a single plasmid that could simultaneously express an RNA and a protein. Such a plasmid would simplify cloning procedures and would reduce the pressure of selection by the use of only one antibiotic. Moreover, it would allow concomitant use of compatible commercial plasmids such as those coding for rare codons (pRARE) commonly used for the production of heterologous proteins in *E. coli*. Two new plasmids, pProRNA and p44K, were thus designed (Figure 1). Plasmid pProRNA was built from the fusion of pACYCT2 and pBSTNAV (see Materials and Methods). It contains the gene encoding for ampicillin resistance. Overexpression of the recombinant RNA is under the control of the constitutive lpp promoter, whereas that of the protein is under the control of the inducible tac promoter. Co-expression assays of the AtRNA with the MS2 coat protein, and of the *E. coli* tmRNA with its partner protein SmpB, using this plasmid are shown in Figure 1. Plasmid p44K is a derivative of the high-copy pUC18 vector. We have replaced the original *lacZ* gene with a bicistronic cassette, which contains the *lac* promoter under the control of the lac operator and the rrnC terminator (Supplementary Figure S1). Plasmid p44K carries the ampicillin resistance gene. Although it does not contain the *lacI* gene, the co-expression of the protein and the RNA before IPTG addition is locked in *E. coli LaqIq* strains like JM101 or DH5 *α* turbo. Figure 1 shows the co-overexpression of the *A. aeolicus* AtmRNA/MS2 coat protein couple. The tmRNA is similarly produced in both plasmids.

As a result, we have designed and constructed three plasmids that allow co-expression of RNA–protein partners in *E. coli* (secondary structures of co-expressed RNA are drawn in Supplementary Figure S2). In our hand, there is no difference in the yield of RNA or protein production according to the plasmids used to achieve the co-production (Figure 1). The applications of such tools are explored in the next paragraphs.

### Co-expression of a tRNA with a modifying enzyme

In our group, we have been studying for many years the annealing process between tRNA^Lys^_3_, the primer of HIV-1 reverse transcription and the viral RNA (26,38,39). We produced the human tRNA^Lys^_3_ in *E. coli* using the pBSTNAV and demonstrated that this recombinant tRNA was active as a primer of the HIV-1 reverse transcription (26). However, certain post-transcriptional modifications, such as the m^1^A_58_, which is crucial in one step of the HIV-1 reverse transcription (40), are lacking because of the heterologous expression in *E. coli.* In this example, it could be convenient to co-produce the enzyme that catalyzes this modification [TrmI, (30,41)] together with tRNA^Lys^_3_. We thus cloned the *T. thermophilus trmi* gene and the DNA sequence coding for the human tRNA^Lys^_3_ into the p44K plasmid. The overexpression of the tRNA^Lys^_3_/TrmI pair in *E. coli* was conducted as described in Materials and Methods. After purification, the methylation state of the tRNA^Lys^_3_ was probed by NMR spectroscopy (Supplementary Figure S3). The success of the co-production *in vivo* with TrmI was evidenced by the comparison of the NMR spectra of tRNA^Lys^_3_ with those of the tRNA overproduced alone in *E. coli*. Indeed, the NMR spectrum of tRNA^Lys^_3_ co-produced with TrmI contains the signal of the m^1^A_58_ methyl and exhibits the chemical shift variation of the imino proton H3 of Ψ_55_ as expected for a tRNA^Lys^_3_ bearing the m^1^A_58_ modification. Therefore, the co-production system is suitable for *in vivo* RNA processing by heterologous enzymes.

### The tRNA–RNA fusion can be produced in MS2 coat protein pseudo-particles

**Figure 2.**
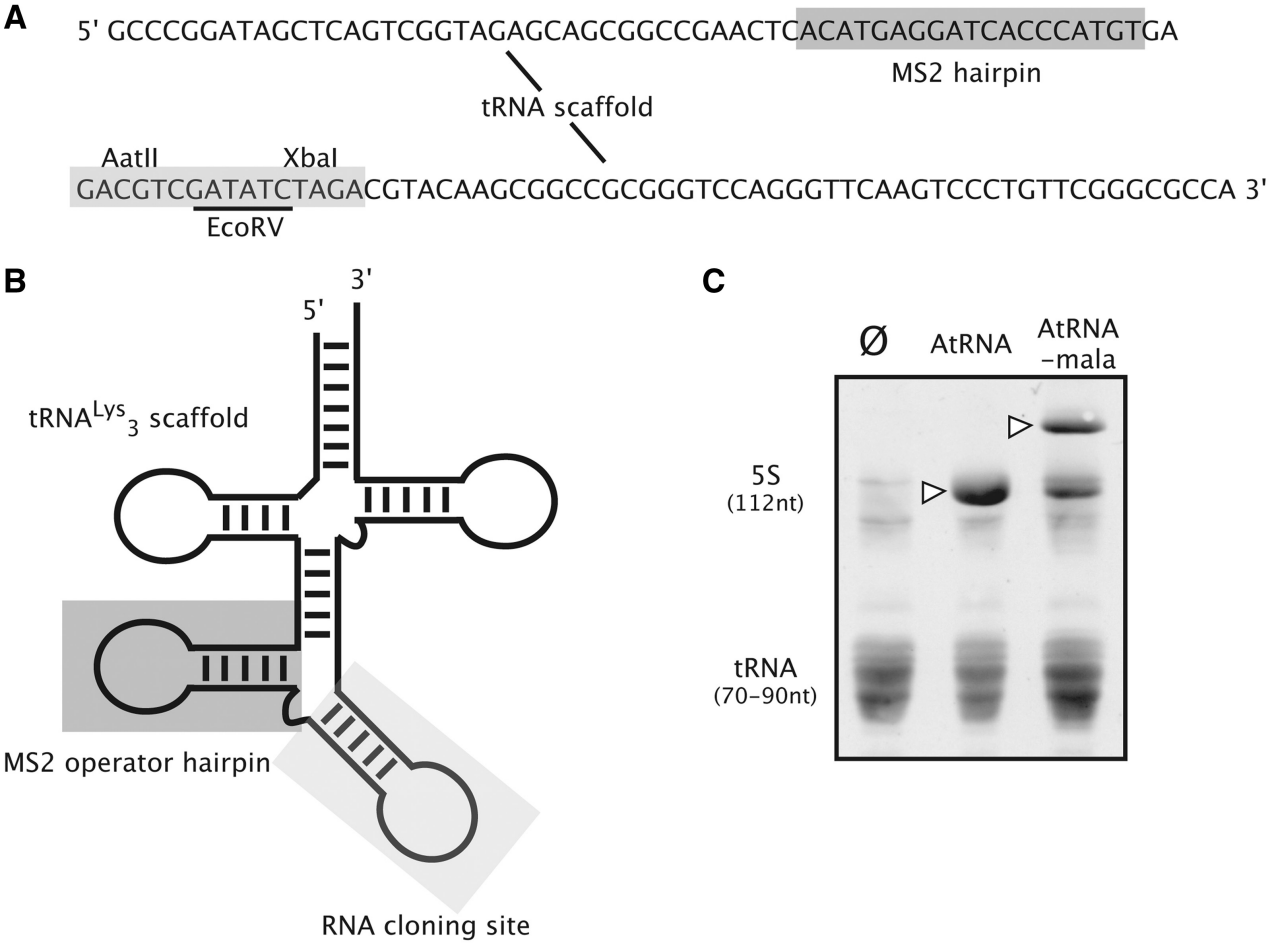
Armored tRNA–RNA fusion. (**A**) Sequences of the DNA inserted into the pBSTNAV-AtRNA plasmid: tRNA^Lys^_3_ scaffold in black, the MS2 operator hairpin boxed in grey, the RNA cloning site boxed in light grey. (**B**) Secondary structure of the corresponding processed transcript using the same color codes. The restriction sites within the RNA cloning site were selected to maintain the hairpin structure. (**C**) Overexpression of AtRNA (123 nt) and AtRNA-mala (155 nt) in *E. coli* JM101 strain. Stroke (control lane): bacteria transformed by the vector with no insert. Crude RNA minipreps were analysed by electrophoresis on a 10% polyacrylamide-urea gel and RNA visualized by UV shadowing. The white triangles indicate the overexpressed RNA.

We have extended our ‘tRNA scaffold’ approach by combining it with the armored RNA technology. This technology comes from the observation that the genomic RNA packaged in the *E. coli* bacteriophage MS2 is resistant to RNase digestion. Bacteriophage MS2, which infects *E. coli,* adopts a simple ribonucleoprotein structure composed of 180 molecules of the MS2 coat protein, one copy of a maturase and one copy of genomic RNA. The MS2 coat protein makes up the bulk of the bacteriophage and assembles into an icosahedral structure of about 26 nm in diameter. Interestingly, pseudo-viral empty particles can be synthesized *in vivo* and *in vitro* from the coat protein alone (42). Moreover, non-phage recombinant RNAs containing an ‘operator’ sequence can be specifically packaged into the pseudo-particles (43). The operator is an RNA hairpin of 19 nt that binds to the MS2 coat protein to initiate the assembly of the bacteriophage particle (44–46). Unlike MS2, which is released into the culture medium upon *E. coli* lysis, armored RNAs remain localized in the cytoplasmic fraction. Our idea was thus to insert the gene coding for this hairpin within the gene coding for the tRNA–RNA fusion (see Figure 2A and B). This AtRNA was successfully produced alone in *E. coli* JM101 strains (Figure 2C).

Next step was to check whether the AtRNA technology could produce functional RNAs. The malachite green aptamer was previously used to probe the functionality of recombinant RNAs produced with tRNA or 5 S rRNA scaffolds (19,21). To use the same functional assay, we inserted a DNA sequence encoding the malachite green RNA aptamer (AtRNA-mala) within the cloning site (Figure 2), which specifically binds triphenyl methane-based dyes, including malachite green (47). The ‘apo’ form of this 34 nt-long aptamer segment adopts an irregular stem-loop structure that changes conformation upon dye binding. We show that AtRNA-mala is successfully overproduced (Figure 2C) and that it efficiently binds to malachite green (Supplementary Figure S4). This indicates that the presence of the MS2 operator hairpin does not impair the overall folding of the recombinant RNA.

**Figure 3.**
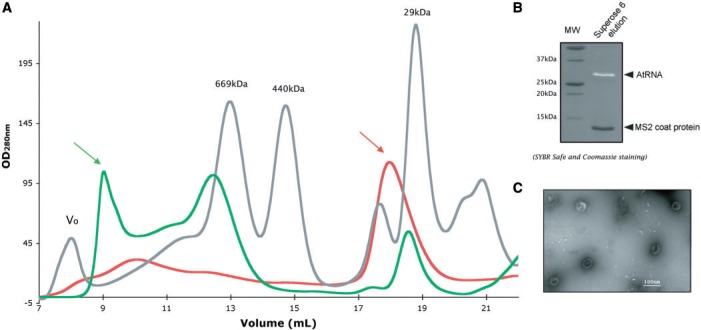
The armored tRNA–RNA fusion is packaged into MS2 coat protein pseudo-particles. (**A**) Size exclusion chromatograms of different supernatants of crude cell extracts of *E. coli* JM101 expression cultures eluted on a Superose 6 column (10/300 GL, GE Healthcare). In red, overexpression of the His6 MS2 coat protein that bears a polyhistidine tag to prevent it from forming pseudo-particles; and in green, overexpression levels of the AtRNA and MS2 coat protein after benzonase treatment of the supernatant. RNA and protein were cloned into the pBSTNAV and the pACYCT2 plasmids, respectively. Arrows indicate the fractions where the MS2 coat protein was found. A chromatogram of reference proteins (thyroglobulin: 669 kDa, ferritin: 400 kDa, carbonic anhydrase: 29 kDa) is indicated in grey. V_0_ is the void volume of the column. (**B**) SDS–PAGE analysis of the fraction denoted by the green arrow in the green chromatogram of Figure 3A. The AtRNA is visualized by SYBR Safe staining (white band, lane 2) and the MS2 coat protein by Coomassie Brilliant Blue staining (black band, lane 2). A molecular weight ladder for protein is given in lane 1. The size of protein standards in kilodalton is given on the left. The black triangles indicate the AtRNA band and the MS2 coat protein band. (**C**) Transmission electron microscopy analysis of the fraction denoted by a green arrow in the green chromatogram of Figure 3A. Images were taken with a screen magniﬁcation of 100 000×.

We then assessed co-production of AtRNA-mala with the MS2 coat protein by inserting the protein gene in the pACYCT2 downstream of the inducible tac promoter (pACYCT2-MS2coat plasmid). *E. coli* JM101 strains were transformed by both plasmids (pACYCT2-MS2coat and pBSTNAV-AtRNA). The expression of AtRNA is constitutive. We tested the induction of the expression of the protein by addition of IPTG, either in exponential or stationary phase of culture growth. The protein was not produced when induced in the stationary phase presumably because the intracellular concentration of AtRNA was maximal during this period. On the contrary, induction in the exponential phase (absorbance at 660 nm around 0.6) proved to be suitable to overexpress both AtRNA and MS2 coat protein. Four hours after induction, the concentration of AtRNA and MS2 coat protein reached 25 and 27 mg/l of culture, respectively. We analysed the co-production of AtRNA and MS2 coat protein obtained in these optimal conditions. After ultrasonicating bacteria, we examined by size exclusion chromatography the supernatant recovered by centrifugation (Figure 3A). As a result, the AtRNA and the coat protein co-elute at a molecular weight >669 kDa (Figure 3A, green chromatogram). This high molecular weight corresponds to the formation of a supramolecular organization consistent with the formation of pseudo-particles. The MS2 coat protein and AtRNA still co-eluate at a high molecular weight, even after extensive benzonase treatment (Figure 3A: green arrow, light green chromatogram; and Figure 3B). These experiments show that co-expression with the MS2 coat protein renders AtRNA completely resistant to RNases under conditions that rapidly degrade naked RNAs (Figure 3A). This observation is consistent with AtRNA being packaged into VLPs. The formation of pseudo-particles by the overproduced MS2 coat protein in the cytoplasm of *E. coli* was observed by electron microscopy as a polydispersed set of vesicles with diameters ranging from 20 to 100 nm (Figure 3C). Altogether, these results demonstrate that we have been able to produce large amounts of recombinant AtRNA–RNA fusion packaged into MS2 coat protein particles and thus resistant to nucleolysis.

### MS2-encapsulated RNA is protected from ribonucleases: example of tmRNA

**Figure 4.**
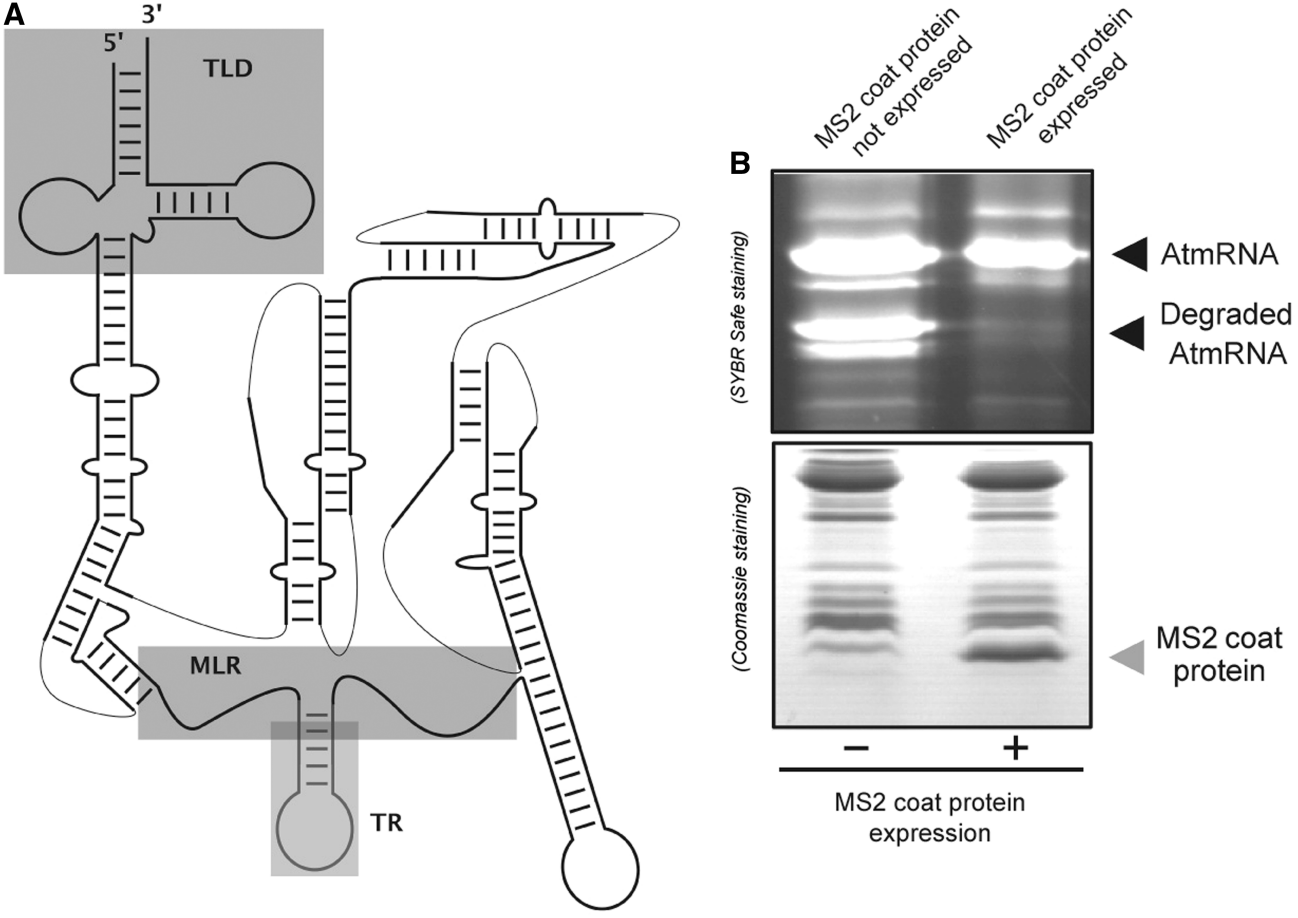
The *A. aeolicus* AtmRNA encapsulated in MS2 coat protein pseudo-particles is protected against nucleases. (**A**) Secondary scheme of *A. aeolicus* AtmRNA showing the MS2 translational operator, the tRNA-like domain and the MLR in grey. (**B**) Overexpression of the AtmRNA alone in *E. coli* JM101 (left part of the upper gel) and co-expressed with the MS2 coat protein (right part of the upper gel): crude RNA minipreps were analysed by electrophoresis on a 10% polyacrylamide-urea gel and RNA visualized with SYBR Safe (upper part). Proteins in the supernatant after bacteria lysis were visualized by SDS–PAGE and Coomassie Brilliant Blue staining (lower gel) before and after IPTG induction (−/+). The black triangle indicates the AtmRNA band and the grey triangle the MS2 coat protein band. The AtmRNA/MS2 coat protein pair was cloned into pBSTNAV and pACYT2 plasmids, respectively.

In our ongoing research on *trans*-translation, we were trying to produce the *A. aeolicus* tmRNA using the pBSTNAV vector in *E. coli*. *Trans*-translation is a highly sophisticated process in bacteria that recycles ribosomes stalled on defective mRNAs and adds a short tag-peptide to the C-terminus of the incomplete polypeptide as degradation signal [for review, see (48)]. The process of *trans*-translation uses the tmRNA, which is a unique molecule with dual tRNA and mRNA functions (Figure 4A). The *A. aeolicus* tmRNA (*A. aeolicus* tmRNA) is a multidomain RNA of 347 nt. It comprises two functional domains, the tRNA domain partially mimicking a tRNA and the mRNA-like region (MLR), which encodes the tag peptide, surrounded by four pseudo-knots. This molecule is, therefore, a case study for our ‘tRNA scaffold’ approach, as it naturally contains a tRNA domain. Although the overproduction is good, the *A. aeolicus* tmRNA undergoes systematic degradation by nucleases during its expression in *E. coli* (Figure 4B). Two bands of approximately the same intensities are observed on a polyacrylamide-urea gel. The lower band evidences a truncated form resulting from the cleavage by nucleases in the cytoplasm of *E. coli* during its overproduction.

We applied the armored RNA technology to tmRNA by inserting the DNA sequence coding for the 19 nt MS2 operator hairpin within the MLR (Figure 4A). We cloned the resulting *A. aeolicus* AtmRNA into pBSTNAV. We transformed the *E. coli* JM101 strains by the pBSTNAV-AtmRNA and the pACYCT2-MS2 coat plasmids. The production of the MS2 coat protein was induced in the exponential phase of the culture for 3 h. The total RNA extracts of bacteria transformed by pBSTNAV-AtmRNA plasmid (lane 1) or co-transformed by the pBSTNAV-AtmRNA and the pACYCT2-coat plasmids (lane 2) are displayed in Figure 4B. As expected, the nucleolytic breakdown that occurs when the *A. aeolicus* AtmRNA is overexpressed alone (Figure 4B) completely disappeared when it was encapsulated by the MS2 coat protein VLP (Figure 4B).

*A. aeolicus* AtmRNA thus presents a nice proof-of-concept for the use of the coupled armored-scaffold technology for the overproduction of large heterologous RNAs. The *A. aeolicus* tmRNA can be purified in a single step by size-exclusion chromatography, either alone following phenol extraction or from the cytoplasmic fraction when encapsulated by MS2.

### Adding a His-tag to the MS2 coat protein prevents formation of pseudo-particles but simplifies the RNA purification protocol

**Figure 5.**
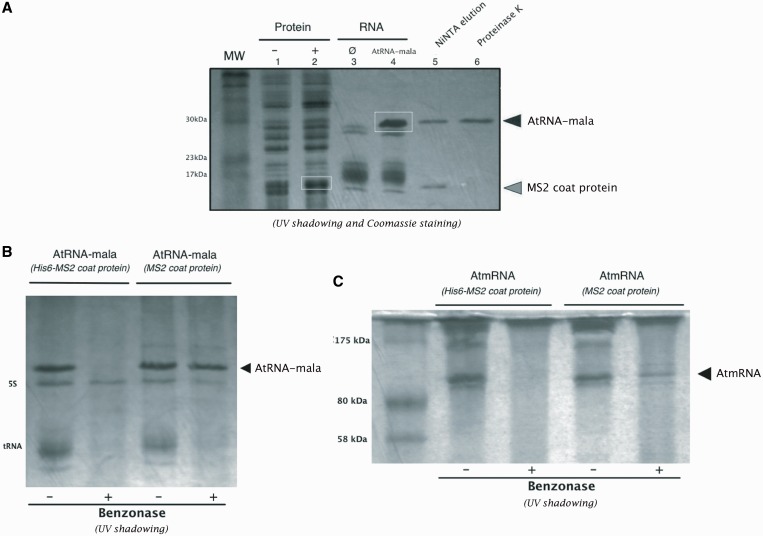
The use of His6-MS2 coat protein simplifies the RNA purification protocol. (**A**) Co-expression of AtRNA-mala/His6-MS2 coat protein in *E. coli* JM101 strain. Crude bacteria extracts before (lane 1) and after (lane 2) IPTG induction, and crude RNA minipreps (lane 3 and 4) were separated on a 16% SDS–PAGE gel and visualized by Coomassie Brilliant Blue staining and UV shadowing. Stroke indicates the control experiment: bacteria transformed by the vector with no insert. White boxes indicate the overexpressed AtRNA-mala and the MS2 coat protein. NiNTA elution (lane 5): the AtRNA-mala/His6-MS2 coat protein complex was eluted in the same fractions upon binding to NiNTA agarose column. The MS2 coat protein can then be digested using proteinase K (lane 6). The black triangle indicates the AtRNA-mala band and the grey triangle indicates the MS2 coat protein band. The molecular weight of protein standards is given in kilodalton on the left. (**B**) Comparison of benzonase resistance of the AtRNA-mala because of co-expression with the MS2 protein (wild-type or His-tagged). RNA extracts in absence (−) or in presence (+) of benzonase in the lysis supernatant (sonication) were analysed by electrophoresis on a 16% SDS–PAGE gel and visualized by UV shadowing. The black triangle indicates the AtRNA-mala. (**C**) Comparison of benzonase resistance of the AtmRNA because of co-expression with the MS2 protein (wild-type or His-tagged). RNA extracts in absence (−) or in presence (+) of benzonase in the lysis supernatant (sonication) were analysed by electrophoresis on a 16% SDS–PAGE gel and visualized by UV shadowing. The black triangle indicates the AtmRNA.

To facilitate the purification of the MS2 coat protein–tRNA-RNA complex, a polyhistidine tag was introduced into the loop region of the MS2 coat protein as previously reported (28). The resulting His6-MS2 construct was co-expressed with either AtRNA, AtRNA-mala or AtmRNA. In all cases, it was noticed that the MS2 coat protein only formed a dimer (Figure 3A, red chromatogram) and not pseudo-particles. Therefore, the polyhistidine tag prevents the MS2 coat protein from assembling into pseudo-particles. Nevertheless, this His6 tag offered a useful way to co-purify the RNA–protein complexes. Figure 5A shows that the AtRNA-His6-MS2 coat protein can be fixed to the Ni-NTA agarose resin and recovered by imidazole gradient elution. Remarkably, the overproduction of the His6-MS2 coat protein prevents the *A. aeolicus* AtmRNA from nucleolysis in *E. coli* despite its inability to form VLPs (Figure 5C).

The ability to purify an RNA–protein complex in a single step demonstrates that the coupling of the tRNA-scaffold and the armored technologies offers interesting perspectives for RNA purification. The simplicity of the method opens the way to parallelization for high-throughput approaches. Usual RNA purification methods begin by phenol extraction followed by ethanol precipitation to remove phenol. Precipitation of large and highly helical RNAs can lead to irreversible formation of aggregates (49). In our protocol, after mechanical disruption, AtRNAs are purified under ‘native’ conditions. By nickel affinity chromatography, we could quickly isolate the AtRNA-mala/His6-MS2 coat protein complex. The MS2 coat protein is then subsequently digested by proteinase K to recover the pure AtRNA-mala (Figure 5A, lane 6). As a result, AtRNA, the *A. aeolicus* AtmRNA and AtRNA-mala were successfully co-purified with the His6-MS2 coat protein using immobilized metal ion affinity chromatography. This step was possible because of the tight interaction between the RNA and MS2 protein. The untagged MS2 coat protein offers greater protection of the target RNA against benzonase activity than its 6-His counterpart (Figure 5B), which is consistent with the formation of pseudo-particles. The wild-type coat protein forms pseudo-particles in solution, whereas the tagged protein exists as a dimer. In both cases, the interaction between the RNA and the protein is maintained. However, only the wild-type protein can protect the RNA from benzonase after extraction.

The possibility to co-purify the AtRNA/His6-MS2 coat protein leads us to investigate if this RNA–protein complex were amenable to X-ray crystallography study (Supplementary Figure S5). Diffracting crystals were rapidly obtained, which we are currently optimizing.

### Co-expressing small regulatory RNA/Hfq in *E. coli* without the tRNA scaffold

**Figure 6.**
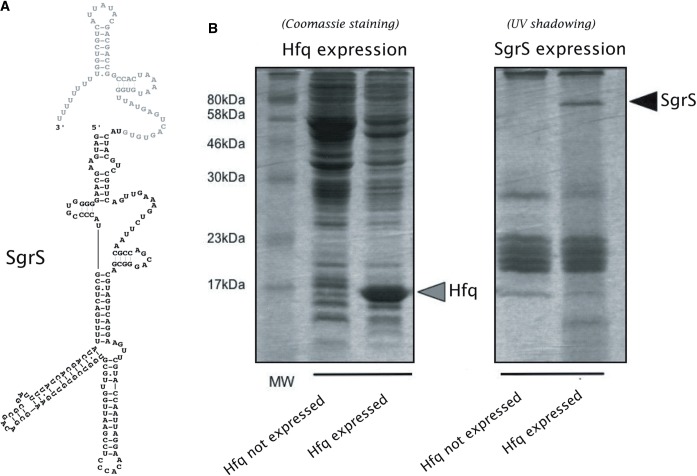
Co-expression of SgrS/Hfq in *E. coli.* (**A**) Predicted secondary structure of *E. coli* K12 SgrS calculated with Mfold (66) showing, in grey, the 3′ portion of SgrS that corresponds to the polyU tail of rho-independent terminator. (**B**) Crude extracts of protein (gel on the left) in absence or presence of Hfq expression (−/+) were analysed by electrophoresis on a 12% SDS–PAGE gel. Crude extract of RNA (gel on the right) in absence or presence of Hfq expression was analysed by electrophoresis on a 12% SDS–PAGE gel. Protein and RNA were visualized by either Coomassie Brilliant Blue staining (left) or UV shadowing (right). The black and grey triangles indicate SgrS band and Hfq, respectively. The SgrS/Hfq pair was cloned in plasmid p44K. Lane 1 (gel on the left) shows protein molecular weight markers and associated molecular masses.

Ultimately, we investigated the possibility of co-expressing RNA/protein pairs without the tRNA scaffold. As the RNA of interest is embedded into and protected by the tRNA scaffold, we considered that an RNA chaperone protein could also play this role. As a proof-of-concept, we co-expressed a target RNA/Hfq pair. The small RNAs associated with the protein Hfq constitute one of the largest classes of post-transcriptional regulators known so far. Together with the extensive list of validated mRNA targets, the regulatory scope of Hfq-associated RNAs has begun to rival that of transcription factors, as illustrated by recently identified new functions in physiological circuits as diverse as biofilm formation (50–52), cell surface modulation (53), amino acid starvation (54,55), sugar import (56,57), quorum sensing behavior (58,59), switch to anaerobic growth (60,61) or virulence factor expression (62). We focussed on SgrS, which is a 227-nt RNA expressed in *E. coli* during glucose–phosphate stress. SgrS negatively regulates translation and stability of the *ptsG* mRNA, which encodes the major glucose transporter. Regulation is achieved via a base-pairing dependent mechanism that requires the RNA chaperone Hfq (63). The PolyU tail of the rho-independent terminator of bacterial small RNAs is essential for Hfq action (64,65) (Figure 6A). We tested the co-production of SgrS with Hfq protein in plasmid p44K. In the cloning procedure (see Materials and Methods), the tRNA scaffold is abolished, and SgrS is thus produced as a stand-alone RNA. The expression of SgrS was observed only when it was co-expressed by Hfq in *E. coli* (Figure 6B). These data show that increasing the production of Hfq decreases the sensitivity of SgrS to nucleolysis and thus confirm the protective role of Hfq on SgrS (Figure 6B). This result opens an effective way to produce any Hfq-associated RNAs in *E. coli*.

## DISCUSSION

In this article, we have developed plasmids for co-expressing RNA–protein couples in *E. coli* and illustrated a number of interesting applications that exploit these new tools. The critical issue for successful RNA–protein co-expression was synchronizing the accumulation of the RNA transcript with the production of the partner protein. This was made possible by a rational choice of promoters that can coexist in the same host cell. We tested several strategies that used either two plasmids (one encoding for the RNA, and the second for the protein) or a single plasmid. We demonstrated that these expression systems can be successfully used by producing several binary RNA–protein complexes and via the efficient purification of the complex from crude extracts by affinity chromatography by means of a fusion tag. Our methods of RNA–protein co-expression can offer attractive methods for expressing proteins that are poorly expressed or incorrectly folded because of the absence of their RNA partner. Our method offers a unique preparative system for overexpressing RNA–protein complexes in *E. coli*, with yields of several milligrams of purified material, which is a prerequisite to structural biology studies. There are a multitude of potential applications of this technology, including RNA production and purification, RNA modifications and the production of RNA–protein complexes. Finally, if needed, the pACYCT2 plasmid can be easily modified to co-express multiple proteins in combination with a recombinant RNA target embedded in a tRNA scaffold.

### RNA production and purification

The first application deals with the production of RNA in large quantities *in vivo*. The *in vivo* expression strategy that we have designed is efficient for expressing structured RNA, at least up to 250-nt long. In addition, our method abolishes the requirement of T7 transcripts with the 5′-end starting with G or GG. However, our previously designed plasmid to produce RNA in *E. coli* has suffered from two limitations: (i) poorly structured or unstructured RNA gets partially or totally degraded and is poorly expressed; and (ii) purification of the RNA requires phenol-extraction steps, which cannot be easily parallelized or transposed to high throughput approaches. Moreover, it is frequently important to work under ‘native’ conditions, especially for large RNA. Phenol extraction often requires ethanol precipitation, which can cause large and highly helical RNAs to form irreversible aggregates.

To address the first issue, we devised an improved chimeric RNA constructs that can be packaged into a phage particle. The only requirement for packaging by RNA phages of the MS2 family was the inclusion of an additional hairpin in the tRNA scaffold. This packaging strategy offers two major advantages: first, upon assembly of the VLPs, the target RNA is rapidly isolated from the cell ribonucleases and is thus protected from degradation; second, RNA-containing pseudo-viral particles are released into the *E. coli* cytoplasm. After cell lysis and VLPs purification by size exclusion chromatography, RNA can be recovered by heat treatment, whereas chemical extraction may decrease the quantity of RNA as described for armored RNA (67). Our strategy is amenable to simple 96-well format parallelization and removes the need for phenol-extraction steps. Consequently, novel combinatorial approaches that focus on RNA can be conceived, for example, genomic library expression or *in vivo* mutagenesis and evolution of RNA structures, followed by screening. Our second proposal to minimize nucleolytic degradation is to co-produce the RNA of interest with a native protein binding partner, for example, an RNA chaperone. We demonstrated that co-production of SgrS with Hfq greatly reduced nucleolysis of the SgrS RNA that occurs in *E. coli* when this RNA is overproduced alone.

### Production of RNA–protein complexes amenable to structural biology

The approach presented in this article constitutes an efficient way to produce milligram quantities of RNA–protein complexes for structural analyses. We showed that we could easily co-express and co-purify the MS2 operator hairpin in the tRNA scaffold and the MS2 coat protein. We also obtained diffracting crystals easily. This work has demonstrated that the RNA–protein co-expression in *E. coli* in the tRNA scaffold is an efficient way to obtain pure and homogeneous RNA–protein complex. This strategy has potential application when studying RNA aptamers that target proteins. The tRNA–RNA-protein co-expression plasmids reported here would be an effective way of producing RNA aptamer–protein complexes for therapeutic and molecular imaging applications or which are directly amenable to structural biology. Our system also has more general applications, for instance an RNA of unknown structure could be inserted into the tRNA scaffold. We have already produced a chimeric RNA, in which the malachite green aptamer is fused to a tRNA scaffold with the MS2 operator hairpin instead of the anticodon loop. This chimeric RNA has been successfully purified via the high affinity interaction between the His-tagged MS2 coat protein and the MS2 operator hairpin. The malachite green aptamer remains functional under these production conditions. Therefore, we can imagine a more general system that would allow the co-purification and the co-crystallization of other RNAs of unknown structure, particularly as our system allows RNA samples to be produced under native conditions without denaturation steps. Furthermore, the use of the tRNA scaffold and MS2 protein would allow the use of molecular replacement to more efficiently determine the 3D structure of the RNA of interest. A further development of this method could be the addition of ribozymes in the tRNA scaffold as previously designed (28) to get rid of the tRNA part after production and purification.

The use of tRNA scaffold for producing RNA targets has already been successfully applied to *in vivo* experiments (17). Thus, our system could also allow *in vivo* monitoring of co-localization of RNA–protein complex, with the possibility of Fluorescence Resonance Energy Transfer (FRET) between a fluorescent RNA and a protein fused with Green Fluorescent Protein (GFP) for instance.

## SUPPLEMENTARY DATA

Supplementary Data are available at NAR Online: Supplementary Figures 1–5.

## FUNDING

Supported by the 6th framework program of the European Union (FSG-V-RNA); the ‘Agence Nationale de la Recherche’ (TriggeRNA and RNAUREUS); the French national research center (CNRS); the University Paris Descartes. Funding for open access charge: Institutional funds or Department fund.

*Conflict of interest statement.* None declared.

## Supplementary Material

Supplementary Data
