# Supplementary material for: Co-expression of RNA–protein complexes in Escherichia coli and applications to RNA biology
Source: Nucleic Acids Res. 2013 Jun 25;41(15):e150. doi: 10.1093/nar/gkt576 (PMC3753655; doi:10.1093/nar/gkt576)
Supplement: Supplementary Data [file supp_41_15_e150__index.html]

Co-expression of RNA–protein complexes in Escherichia coli and applications to RNA biology — Co-expression of RNA–protein complexes in Escherichia coli and applications to RNA biology — Supplementary Data 

# Co-expression of RNA–protein complexes in *Escherichia coli* and applications to RNA biology

## 

files

**Files in this Data Supplement:**

- Supplementary Data - doc file
