# Supplementary material for: Co-expression of RNA–protein complexes in Escherichia coli and applications to RNA biology
Source: Nucleic Acids Res. 2013 Jun 25;41(15):e150. doi: 10.1093/nar/gkt576 (PMC3753655; doi:10.1093/nar/gkt576)

**Supplementary Figure 1.** Sequences of the cloning and expression regions are given for each plasmid. The pBSTNAV and pACYCT2 plasmids are used to co-express RNA/protein pairs using the two-plasmid strategy. pACYCT2 differs from pACYCDuet-1 (Novagen) by substitution of the T7 promoter for a tac promoter in the second multiple cloning site. The sequence of the cloning region is detailed in the lower part of the figure. pBSTNAV is a shorter plasmid that gives good overexpression of RNA (23). The pBSTNAV and the pACYCT2 have different antibiotic resistance genes and two different replication origins and are therefore compatible. The p44K plasmid contains a tRNA like scaffold (nucleotides in grey).

**Supplementary Figure 2.** Secondary structures of RNA produced for this study.

**Supplementary Figure 3.** Co-expression of a tRNA m^1^A_58_ methyltransferase (TrmI) with a tRNA lacking this modification allows us to overproduce a tRNA bearing an m^1^A_58_ nucleotide. Superimposition of NMR NOESY experiments showing the region encompassing the imino groups (10-12 ppm) and the methyl groups (1-2 ppm). Standard over production of tRNA^Lys^_3_ in *E. coli* with an unmodified A_58_, in black; co-expression of tRNA^Lys^_3_ with TrmI in *E. coli*, in red; and *in vitro* methylated tRNA^Lys^_3_ purified and then methylated *in vitro* by TrmI thus bearing a m^1^A_58_ nucleotide. The tRNA^Lys^_3_/TrmI couple was cloned into the p44K plasmid.

After purification, the methylation state of the tRNA^Lys^_3_ was probed by NMR spectroscopy (red spectrum). The success of the co-production *in vivo* with TrmI was evidenced by the comparison of the NMR spectra of tRNA^Lys^_3_ with those of the tRNA overproduced alone in *E. coli* (black spectrum) or overproduced alone in *E. coli* and then modified *in vitro* by TrmI (green spectrum). The red spectrum contains the signal of the m^1^A_58_ methyl and exhibits the chemical shift variation of the imino proton H3 of Ψ_55_ as expected for a tRNA^Lys^_3_ bearing the m^1^A_58_ modification. However, the doubling of the signal of Ψ_55_ discloses the presence of a second species corresponding to the non-modified tRNA^Lys^_3_. Nevertheless, the m^1^A_58_ modification was succesfully incorporated into the recombinant tRNA, but at a substoichiometric level. The co-production system is therefore suitable for *in vivo* RNA processing by heterologous enzymes.

**Supplementary Figure 4.** Malachite green aptamer function. A. Left part: the interaction between malachite and the aptamer modifies the absorbance of the dye. Right part: Absorbance spectra of malachite green (green trace), malachite green with a non specific RNA chimera (tRNA^Lys^ / HBV epsilon, black trace) and malachite green complexed with the specific aptamer RNA chimera (red trace). The same concentration of the dye (5 μM) was used in each experiment. B. Left part: Redshift of the malachite green fluorescence in the presence of the aptamer RNA chimera: left cell, malachite green, right cell, malachite green complexed with the RNA chimera. Right part: Fluorescence emission spectra of malachite green alone (green trace), in presence of a non specific RNA chimera (tRNALys / HBV epsilon, black trace) and of the specific aptamer RNA chimera (red trace). Concentration of the dye is the same in all three experiments.

**Supplementary Figure 5.** Evaluation of the amenability of the AtRNA/His6-MS2 coat protein couple to X-ray crystallography. (**A**) Size exclusion profile of the AtRNA/6-His MS2 coat protein pair and SDS-PAGE gel (12%) analyzing the fraction with the maximum absorbance at 280 nm. This step of purification was carried out after a Ni-NTA agarose column. (**B**) left: diffracting crystals obtained from a sample of AtRNA/His6-MS2 coat protein couple concentrated to 4 mg/mL. Right: One image of diffraction data collected on beamline ID23-2 (European Synchrotron Radiation Facility, Grenoble, France).

Supplementary Figure 1


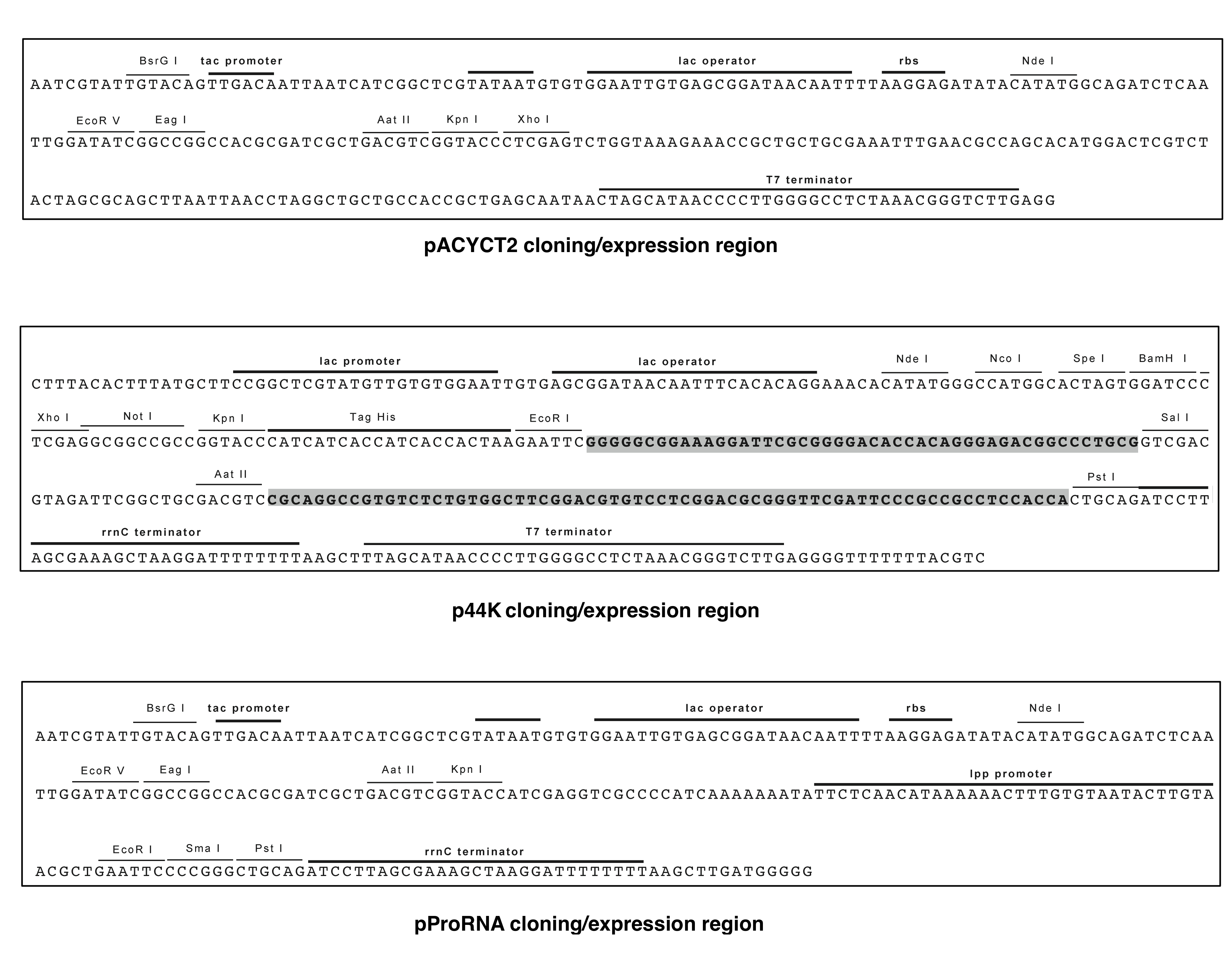


Supplementary Figure 2


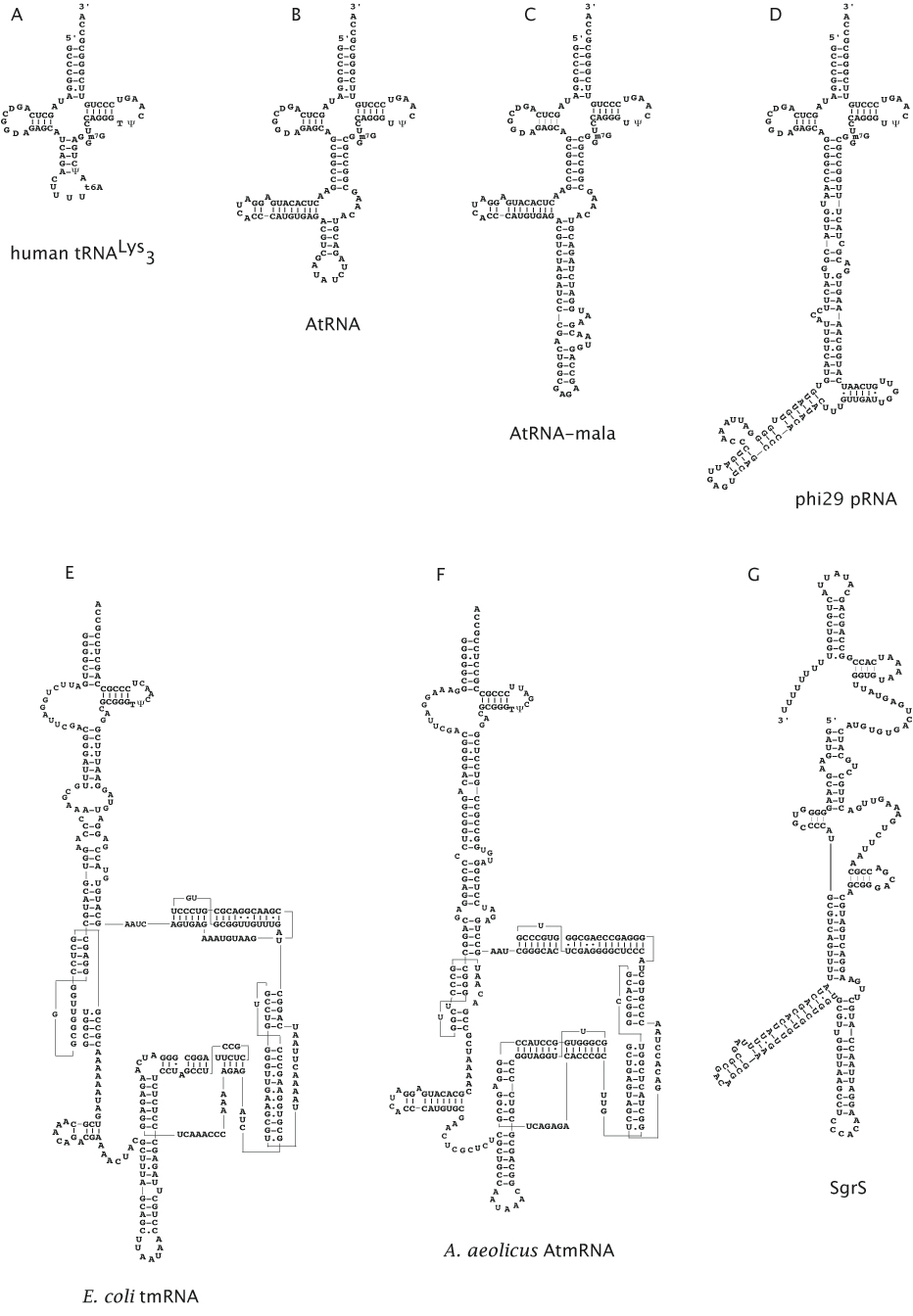


Supplementary Figure 3


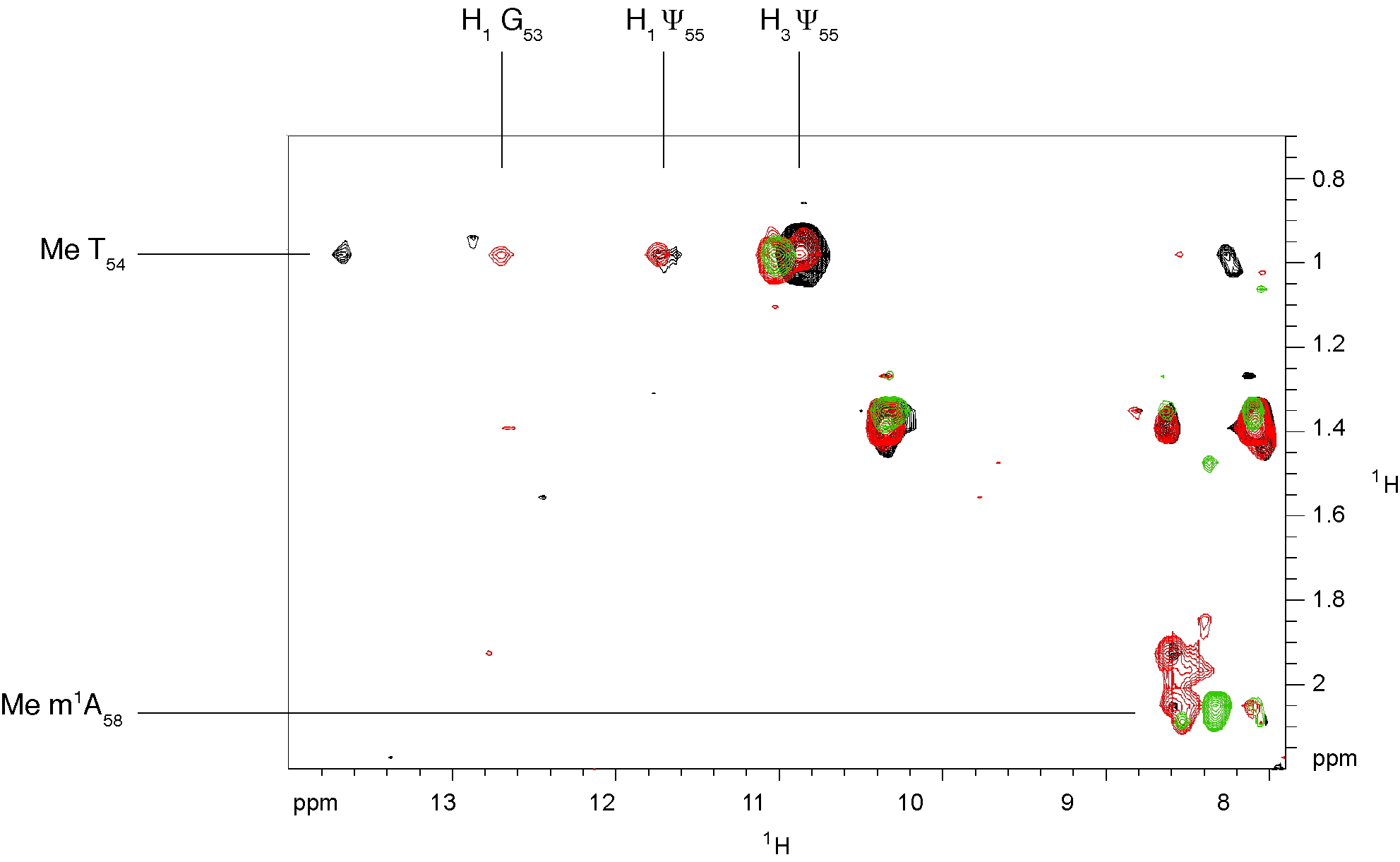


Supplementary Figure 4


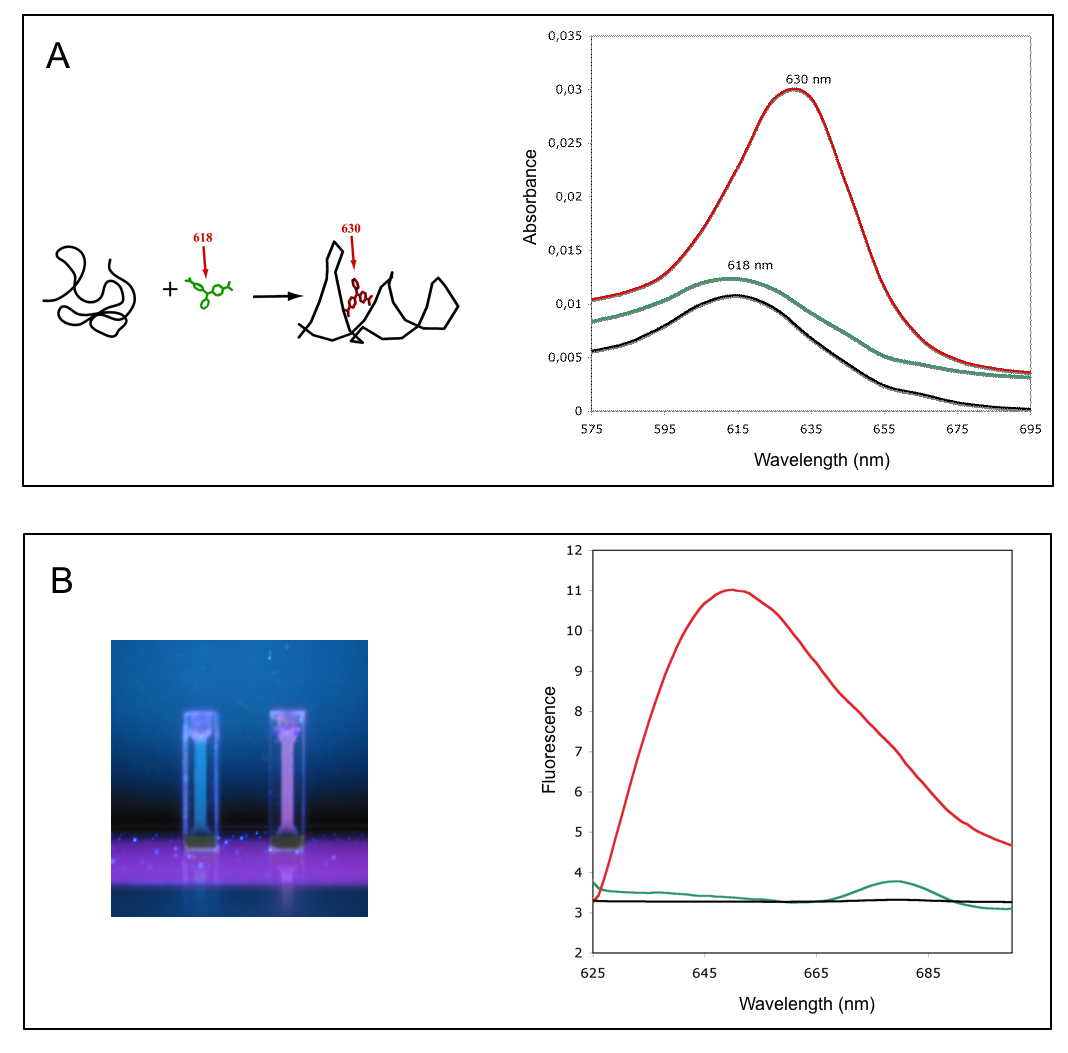


Supplementary Figure 5


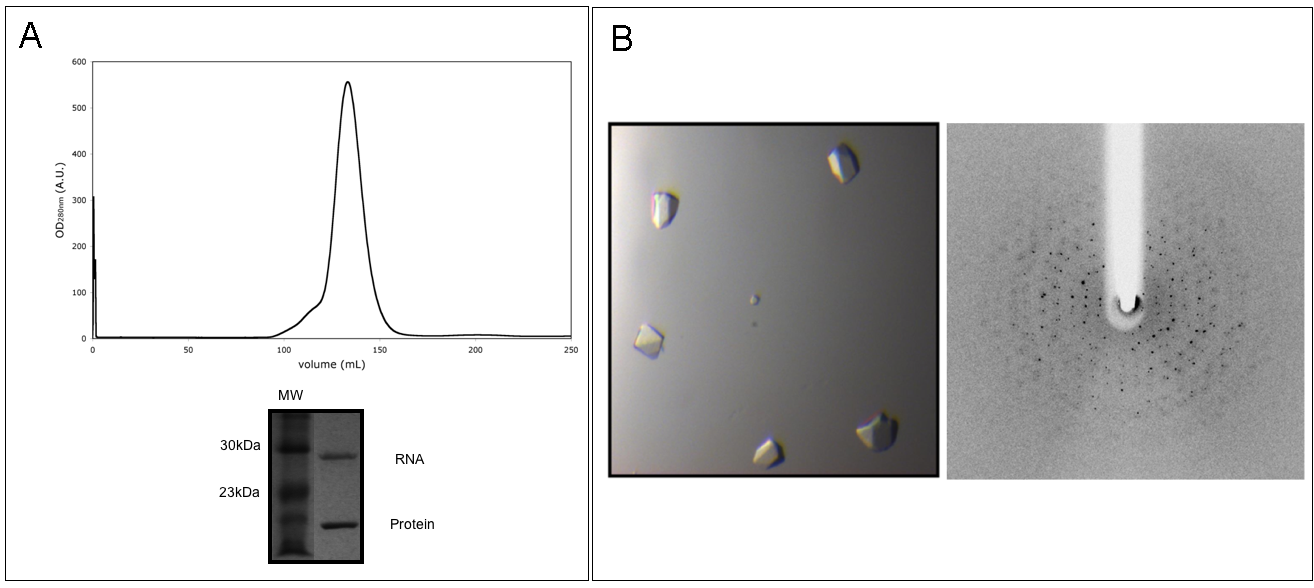

Supplement: Supplementary Data [file supp_gkt576_nar-01247-met-h-2013-File002.doc]
